# Coding-complete genomic sequence of a rhinovirus C-32 in a human nasal swab sample that tested false positive in a SARS-CoV-2 antigen test

**DOI:** 10.1128/mra.00172-24

**Published:** 2024-03-25

**Authors:** Tracey L. Moquin, Kuttichantran Subramaniam, John A. Lednicky

**Affiliations:** 1 Department of Environmental and Global Health, College of Public Health and Health Professions, University of Florida, Gainesville, Florida, USA; 2 Emerging Pathogens Institute, University of Florida, Gainesville, Florida, USA; 3 Department of Infectious Diseases and Immunology, College of Veterinary Medicine, University of Florida, Gainesville, Florida, USA; Queens College Department of Biology, Queens, New York, USA

**Keywords:** rhinovirus, COVID-19, COVID-19 rapid antigen test

## Abstract

Rhinovirus-A was previously shown to cause false-positive results in a Japanese SARS-CoV-2 antigen test. We report that a false-positive result was obtained in a specimen with rhinovirus C-32 that had been tested using an American SARS-CoV-2 antigen test.

## ANNOUNCEMENT

**Fig 1:**
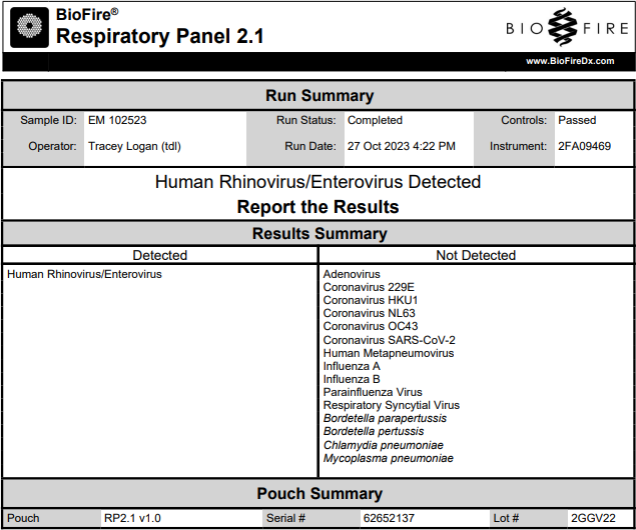
Data readout from Biofire multiplex PCR FilmArray Respiratory Panel 2.1 test.

The gold standard for COVID-19 testing has been the detection of severe acute respiratory syndrome coronavirus 2 (SARS-CoV-2) RNA through reverse transcriptase polymerase chain reaction (RT-PCR). But now that the COVID-19 pandemic has been downgraded by the WHO to a public health emergency of international concern, rapid at-home lateral flow antigen tests are typically used due to their widespread availability and ease of use. A nasal swab specimen obtained from a person with acute respiratory illness tested positive using an Abbott BinaxNOW COVID-19 Antigen Self-Test at a local clinic on 25 October 2023. To analyze the SARS-CoV-2 variant, a separate nasal swab specimen was obtained the same day using a flocked nylon swab, which was immersed into universal transport medium (UTM) (COPAN Diagnostics, Inc., Murrieta, CA, USA). However, RT-PCR tests targeting the *N*- and *RdRp* genes (1) of SARS-CoV-2 RNA in nucleic acids extracted using a QIAmp Viral RNA Mini Kit (QIAGEN Sciences, Inc., Germantown, MD) from material extruded into the UTM generated negative results. Attempts to isolate a virus in replicates of LLC-MK2, HeLa, MRC5, and Vero E6 cells (1, 2) inoculated with aliquots of the material in UTM and incubated at 33°C and 37°C for an observation period of 30 days were unsuccessful. An aliquot of the sample in UTM was then analyzed using a Biofire multiplex PCR FilmArray Respiratory Panel 2.1 test (Bio Mérieux, Marcy‐l'Etoile, France), which detected Rhinovirus/Enterovirus (Fig. 1). To identify the specific virus, nucleic acids that had been extracted from the sample were processed for next-generation sequencing. This work was IRB exempt, as this was a one-time assessment from a single person.

A cDNA library was generated using a NEBNext Ultra RNA Library Prep Kit (New England Biolabs) and sequenced on an Illumina NextSeq1000 platform (Illumina, Inc., San Diego, CA). Upon completion of the sequencing run, a total of 95,207,899 reads with an average read length of 215  bp were obtained, and 85% of the human host sequences (GenBank accession number MSBL00000000.1) were removed using Kraken v2.0 (3). D*e novo* assembly of the remaining untrimmed paired-end reads (14,650,757) was performed using MEGAHIT v1.1.4 with default parameters (4). BLASTX searches of the resulting contigs, using OmicsBox v1.2 against the National Center for Biotechnology Information nonredundant protein database, revealed a human rhinovirus C-32 (RV-32) (family Picornavirida*e,* genus *Enterovirus*, species *Rhinovirus C*) genome with an average coverage of 445 reads/nucleotide. Whereas the RV-32 coding region sequences were obtained, the 5′ and 3′ non-translated region sequences were not verified using rapid amplification of cDNA ends, so the genomic sequence we determined is considered “coding-complete” instead of “complete.”

The coding-complete RV-32 genome of this work has a G/C content of 42.70%, and a nucleotide BLAST search revealed 91% identity (6,423/7,034 nt) with that of RV-32 strain USA/CA/RGDS-2016-1008 (GenBank MK520815.1). A Protein BLAST search indicated that the deduced polyprotein amino acid sequence has 99% (2,111/2,142) identity with that of USA/CA/RGDS-2016-1008 (GenBank QBM01046). We designated the rhinovirus C genome that we analyzed as RV-32/USA/FL/UFEPI-2023-1, and its nucleotide sequence was deposited in GenBank (accession no. GenBank PP314216).

## Data Availability

The genome sequence and raw sequence data for human rhinovirus C-32 (RV-32/USA/FL/UFEPI-2023-1) have been deposited in the NCBI GenBank and Sequence Read Archive (SRA) databases under accession no. PP314216.1 and SRR28013887, respectively.
